# Editorial: Exploring the interplay between clinical and non-clinical outcomes for children and adults with inflammatory bowel disease

**DOI:** 10.3389/fgstr.2023.1311951

**Published:** 2023-11-13

**Authors:** Angharad Vernon-Roberts, Tiffany Taft, Taryn Lores, Jospeh Meredith, Christian P. Selinger

**Affiliations:** ^1^ Department of Paediatrics, University of Otago Christchurch, Christchurch, New Zealand; ^2^ Oak Park Behavioral Medicine LLC, Oak Park, IL, United States; ^3^ Royal Adelaide Hospital, Central Adelaide Local Health Network, Adelaide, SA, Australia; ^4^ School pf Psychology, Deakin University, Melbourne, VIC, Australia; ^5^ Calvary Paediatrics, Canberra, ACT, Australia; ^6^ Department of Gastroenterology, Leeds Teaching Hospitals NHS Trust, Leeds, United Kingdom; ^7^ Leeds Institute of Medical Research at St James’s University Hospital, University of Leeds, Leeds, United Kingdom

**Keywords:** inflammatory bowel disease, disease outcomes, pediatric, resilience, quality of life, stigma, psychosocial

Inflammatory bowel disease (IBD) is a chronic immune-mediated condition of the gastrointestinal tract that can affect both children and adults. People with IBD often experience a significant symptom burden and undergo complex treatment plans that can negatively affect their physical, mental, and social well-being. The clinical outcomes of IBD are frequently studied, as are multifactorial non-clinical outcomes such as treatment adherence, resilience, self-management, knowledge, and quality of life (QoL). In contrast, research focusing on the interplay between clinical and non-clinical outcomes is minimal. This topic deserves greater attention to identify potential positive or negative interactions, and modifiable factors that could be targeted in IBD management. The goal of this Research Topic was to present the latest research and reviews that study the interplay between clinical and non-clinical outcomes in IBD. This research advances our understanding of the importance of holistic care provision whereby all aspects of an individual’s health are addressed not just clinical parameters.

Despite many medical advances in IBD treatment and practice patterns in recent decades, surgical interventions remain indicated for those with intractable disease, or complications (1–4). In addition, in some settings such as isolated terminal ileal Crohn’s disease (CD), surgery provides sustained benefits equal to anti-tumor necrosis therapy [biologics] (5). IBD surgery may significantly impair patients’ QoL, social participation, productivity, and psychosocial outcomes (4). Conversely, QoL among adults with CD has been shown to improve immediately after surgery and longitudinally (6). However, little research on this topic has been undertaken with the pediatric IBD population, a gap which was addressed by Dipasquale et al. in this Research Topic. This research showed that QoL was low in the year prior to surgical intervention but increased significantly following surgery in all QoL dimensions. Also, there were improvements in many IBD symptom domains, and activities of daily living. School absences significantly decreased following surgery, social functioning increased, and feelings of anger, injustice and embarrassment improved. However, no difference was seen in the domain of concern for future health problems. These results highlight that those with severe disease activity requiring surgery may experience greater physical and psychological well-being due in part to alleviation of symptoms.

All children diagnosed with IBD will eventually have to transition from the pediatric to adult healthcare setting. This transition process involves the development of health autonomy, self-management skills, communication skills, assertiveness, and decision-making (7). The transition period is often associated with adverse health outcomes due to reduced adherence to management regimens (8–11). In particular, psychosocial wellbeing is at risk, as transition occurs at a time when adolescents are experiencing many physical, mental, and developmental changes. Mendiolaza et al. report in this Research Topic the value of adopting a psycho-gastroenterology approach in adolescent IBD care, based on previous research that delineated adolescent perceived barriers and facilitators to IBD transition. They outline five interventions/strategies that may be implemented to help adolescents through the transition process. These include developing a disease narrative, practicing gratitude, paying it forward by helping within the IBD community, setting goals to achieve tasks, and mastering the brain-gut axis. The review provides guidance for gastroenterologists and other health care professionals to effectively support patients to develop their adult patient identity.

IBD is often considered to be a concealable or invisible disease as individuals may not outwardly appear sick. Disease invisibility may in turn lead to insensitivity by others due to poor understanding of the condition and the needs of those with IBD (12, 13). Up to 84% of people with IBD report perceived stigma, whereby they believe a social stereotype is being held against them (14, 15). Perceived stigma is known to reduce treatment adherence, self-efficacy, and health-related quality of life, and increase anxiety and depression (14, 16). Conversely having resilience, defined as achieving positive outcomes in the face of adversity or risk (17), is associated with positive outcomes such as body image, social functioning, and quality of life (18–20). In this Research Topic, Lenti et al. reported on the interplay between stigmatization, resilience, and disease activity. Their findings showed that higher resilience levels were inversely associated with disease activity level and levels of perceived stigma, suggesting a mediating relationship. In addition, inverse correlations were found between high levels of stigma and lower self-efficacy and self-esteem, suggesting that these factors also play an important role in preventing stigmatization. Perceived stigma may therefore be a modifiable factor worth targeting in adults and children with IBD, at the individual level and for the wider community (21, 22).

Children diagnosed with IBD before the age of ten years have been shown to have a more severe disease course and a higher rate of resistance to immunosuppressive treatment (23–26). A retrospective review by Krauthammer et al. in this Research Topic reported on the variable longitudinal outcomes seen for infants diagnosed with IBD. The cohort in the study showed favorable remission rates compared to previous research, but still only 73% had achieved longitudinal remission after a median follow-up of 51 months. During this follow-up time, whereby children in the study had not yet reached the age of five years, 26% had undergone IBD surgery due to severity of their disease, and 50% were steroid dependent. Such outcomes pose a substantial challenge during the first years of life: these children face a prolonged and significant burden of disease during a time of rapid growth, and physical and mental development. Further research should be carried out to study the long-term effects of early medical intervention on outcomes such as psychological well-being and comorbidity.

While psychosocial factors have a known association with IBD, external mediating influences may represent an additional burden and may affect people with IBD disproportionately compared to their healthy peers. The global coronavirus pandemic (COVID-19) affected the psychosocial outcomes of those with, and without, the virus due to widespread illness, mortality, and mandated lockdown/isolation policies. In this Research Topic, Zhang et al. studied psychosocial function among children with IBD during the COVID-19 pandemic. They found that for the cohort overall before the pandemic, higher disease activity (measured by CRP level) was strongly associated with daytime dysfunction as related to sleep quality, and interpersonal problems. Longer disease duration was associated with poor sleep quality, interpersonal problems, and panic/agoraphobia, suggesting that those with a longer disease course may be susceptible to maladjustment during stressful events. When results from before and during the pandemic were compared, positive improvements were seen for QoL and sleep quality, negative mood and feelings of ineffectiveness reduced, and no changes in depression or anxiety scores were found. These results highlight that the pandemic did not negatively affect the psychological well-being of children with IBD and in fact may have been beneficial. However, rates of self-reported anxiety and depression symptoms among parents/guardians of children with IBD were significantly higher than those of healthy children during the pandemic, mainly stemming from concerns about their child’s IBD and not being to seek medical treatment.

It is hoped that in highlighting some of the recent research on the relationship between clinical and non-clinical outcomes for people with IBD, care settings can be encouraged to be more holistic in their approach. Future research should include a component of both outcomes to help assess how they may influence each other. This research highlights several psychosocial outcomes that are modifiable, and efforts should be made to explore this further.

## Author contributions

AV-R: Conceptualization, Project administration, Writing – original draft. TT: Conceptualization, Project administration, Writing – review & editing. TL: Conceptualization, Project administration, Writing – review & editing. JM: Conceptualization, Project administration, Writing – review & editing. CS: Conceptualization, Project administration, Writing – review & editing.
